# Therapeutic Treatment With OLX‐07010 Inhibited Tau Aggregation and Ameliorated Motor Deficits in an Aged Mouse Model of Tauopathy

**DOI:** 10.1111/jnc.70025

**Published:** 2025-03-07

**Authors:** E. J. Davidowitz, P. Lopez, D. Patel, H. Jimenez, A. Wolin, J. Eun, L. Adrien, J. Koppel, D. Morgan, P. Davies, J. G. Moe

**Affiliations:** ^1^ Oligomerix, Inc. White Plains New York USA; ^2^ Oligomerix, Inc. Bronx New York USA; ^3^ The Litwin‐Zucker Research Center for the Study of Alzheimer's Disease The Feinstein Institutes for Medical Research, Northwell Health Manhasset New York USA; ^4^ Department of Translational Neuroscience and the Alzheimer's Alliance Michigan State University Grand Rapids Michigan USA

## Abstract

Targeting tau protein is a strategy for the development of disease‐modifying therapeutics for Alzheimer's disease (AD) and numerous rare tauopathies. A small molecule approach targeting tau aggregation was used to select and optimize compounds inhibiting tau self‐association in vitro that have translated in vivo in preventive studies in htau and P301L tau JNPL3 mouse models of tauopathy. In this therapeutic treatment study, aged JNPL3 mice with pre‐existing tau aggregates were used to evaluate the therapeutic effect of OLX‐07010. The study had a Baseline group of mice aged 7 months, a vehicle, and two dose groups treated until 12 months by administration in feed. The primary endpoint of the study was the reduction of insoluble tau aggregates with statistical significance. The secondary endpoints were dose‐dependent reduction of insoluble tau aggregates, reduction of soluble tau, and improvement of motor behavior. ELISAs and immunoblots were used to determine the levels of tau and its aggregated forms including self‐associated tau and Sarkosyl insoluble tau. Effect on motor behavior, as measured by Rotarod assay, was also assessed between the treatment groups. At the end of treatment, reduced levels of self‐associated tau, Sarkosyl insoluble tau aggregates, and overall levels of tau in the heat‐stable fraction with statistical significance in the cortex were observed. Treatment prevented the accumulation of tau aggregates above baseline, and in parallel, treatment groups had improved motor behavior in a Rotarod assay compared to baseline and vehicle control groups, suggesting that treatment was rescuing motor impairment in aged mice. The functional and biochemical readouts suggest that this small molecule has potential for treating neurodegenerative diseases characterized by tau aggregation such as AD and progressive supranuclear palsy.

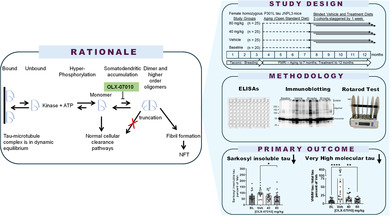

## Graphical abstract

Tau protein aggregates are a common pathological feature of Alzheimer's disease and numerous rare neurodegenerative diseases, such as progressive supranuclear palsy (PSP) and frontotemporal dementia (FTD), collectively called tauopathies. We discovered a small molecule OLX‐07010 that was found to prevent the accumulation of tau aggregates in studies of mouse models of tauopathy treated from a young age. This study evaluated whether treatment with OLX‐07010 of aged mice with pre‐existing tau pathology could have a therapeutic effect as patients are usually treated after the onset of disease. Treatment was provided by mixing OLX‐07010 into the mouse diets. The study showed that treatment prevented the progressive accumulation of tau aggregates and improved their motor coordination that usually becomes impaired in these mice as they age. These results suggest that this small molecule has potential for treating neurodegenerative diseases characterized by tau aggregation.

**Abbreviations**
AD: Alzheimer's disease
AUC: area under curve
BL: baseline
FIMR: Feinstein Institutes for Medical Research
HRP: horse radish peroxidase
HS: heat stable
htau: human tau
IACUC: Institutional Animal Care and Use Committee
mAb: monoclonal antibody
RRID: Research Resource Identifier
Veh: vehicle
VHMW: very high molecular weight

## 1 Introduction

Orally available, small molecule disease‐modifying therapeutics for Alzheimer's disease (AD) are needed to effectively treat large populations over diverse geographic areas. Aberrant posttranslational modification, localization and aggregation of tau protein have been linked to the progression of AD and its reproducible spread through synaptically connected regions in the brain suggesting that targeting tau may be efficacious in treating AD. OLX‐07010 is a clinical stage small molecule from a CNS druglike series of compounds selected for inhibiting full length tau self‐association into dimers and inhibiting monomer addition into larger aggregates. A primary advantage of Oligomerix's approach is that compounds target tau upstream of its misfolding and aggregation, which are highly selective. Ongoing nonclinical evaluation has shown a favorable profile for OLX‐07010 in in vitro pharmacology assays, panels for off‐target activity, and animal studies. Approaches targeting beta sheet structures that form during fibril formation may lead to selecting molecules with off‐target effects, as beta sheet structures are generally found in proteins. OLX‐07010 is a heterocyclic molecule, less than 400 Da in size, that was able to enter the brain following oral administration in a pharmacokinetic study of OLX‐07010 in w.t. mice (Figure S1; Tables S1 and S2). Treatment of young human tau (htau) and P301L tau JNPL3 mouse models of tauopathy, representing tau aggregation in AD and 4R tauopathies mice from 3 to 7 months of age prevented the accumulation of tau aggregates (Davidowitz et al. 2023, 2020).

A disease‐modifying therapeutic (DMT) should block the progression of tau‐mediated neurodegeneration for the treatment of patients after the onset of the disease. Therefore, this study used a therapeutic treatment paradigm to evaluate the effect of treatment in mice following the onset of tau pathology from 7 to 12 months of age in JNPL3 mice. The highest dose in this study was doubled from 40 mg/kg/day in the preventive treatment study to 80 mg/kg/day to achieve a therapeutic effect. Another novel aspect of this study was the evaluation of the effect of treatment on hind limb motor impairment that develops in aged JNPL3 mice (Lewis et al. 2000).

## 2 Methods

### 2.1 Animals

Female homozygous P301L tau JNPL3 mice (Lewis et al. 2000); Research Resource Identifier (RRID): IMSR_TAC:2508; Stock Tg (Prnp‐MAPT*P301L)JNPL3Hlmc were purchased from Taconic Biosciences (Rensselaer, NY). The rationale for using females includes their more aggressive and less variable phenotype compared to male mice (Buccarello et al. 2017; Koppel et al. 2019), which can lead to more pronounced and measurable effects of the drug, making it particularly useful in early‐stage studies to detect potential therapeutic benefits more clearly. Additionally, using female JNPL3 mice maintains consistency with previous research with OLX‐07010 in the preventive study in JNPL3 mice, allowing for more direct comparisons and validation of results. A preventive treatment study with OLX‐07010 in htau mice showed it was effective in males, as well as not being dependent on sex (Davidowitz et al. 2023, 2020). Ideally, highly powered studies including males and females should be performed to evaluate translational potential (Dennison et al. 2021) when feasible. A study performed in male JNPL3 mice had a treatment duration of 6 months from 9 to 15 months of age that required a sample size of 49 male mice per group to show an effect (Okuda et al. 2015). The mice were received at Feinstein Institutes for Medical Research (FIMR; Manhasset, NY) at 2 months and aged to 7 months prior to the start of treatment. Mice were examined, handled, and weighed prior to the initiation of the study and examined at least once a week during treatment to ensure adequate health and suitability. As recommended by The Guide for the Care and Use of Laboratory Animals, rooms were maintained with 12 h light/dark cycles, at a temperature of 20°C–23°C with a relative humidity of approximately 50%. Four mice were housed per cage in standard cages with access to food and water *ad libitum* for the duration of the study. This study did not use environmentally enriched housing. For all mouse cages, adequate nesting material was provided in addition to the usual cage bedding to alleviate thermal stress and improve overall welfare during the treatment period. Mice were euthanized by cervical dislocation under deep anesthesia induced by an overdose of isoflurane (3%–5%), during the middle of the dark cycle. All experiments were conducted in compliance with the FIMR Institutional Animal Care and Use Committee (IACUC; Protocol # 2017‐022).

### 2.2 Mouse Diets

Open Standard Diet (Research Diets; New Brunswick, NJ) was used for aging the mice to 7 months and for the Vehicle group. The diet was formulated with the test article OLX‐07010 manufactured by Albany Molecular Research Inc. (AMRI, Albany, NY); its purity was > 99% by HPLC (AUC). Diets were formulated with 200 or 400 mg of OLX‐07010 per kg of diet for the 40 and 80 mg/kg mouse dose treatment groups based on average weight and daily consumption. No dyes were used in diet formulations. Diets were assayed in duplicate to qualify the levels and stability of the compound in the feed by LC–MS/MS (Quintara Discovery, Hayward, CA).

### 2.3 Bioanalytical Analysis

Quantification of compound levels in mice sera was performed in‐house using LC/MS/MS (Agilent 6400 LC/TQ). Calibration standards of OLX‐07010 were prepared by spiking the compound into blank serum at a series of concentrations. Twenty microliters of the samples or standards were treated with 100 μL of internal standard (verapamil) in methanol to precipitate the protein. The mixtures were vortexed for 15 min and centrifuged at 4000 rpm at 4°C for 15 min. Ten microliters of the supernatant were transferred to an injection plate and mixed with 140 μL of methanol for analysis via positive electrospray UHPLC/MS/MS in multiple‐reaction‐monitoring mode. This method has been qualified for the detection and quantification of our lead compound through evaluations of its specificity, selectivity, linearity, accuracy, precision, recovery, carryover, and stability.

### 2.4 Motor Behavior

Motor function was assayed with MazeEngineers 6‐Lane rotarod for mice (Deacon 2013; Koppel et al. 2019). All mice were acclimated 2 weeks prior to the experimental trials on the device at four rotations/min (r/min) on the 3 cm rotating rod for 10‐min sessions. For experimental trials, six mice were run at a time on lanes 6 cm in width, with rotations that began at a start speed of 4 r/min and then accelerated at 8 r/min to a maximum speed of 20 r/min for either 30 min or until the mice dropped from the rotational drum at a drop height of 16 cm. Utilizing infrared floor sensors that detect true falls, integrated Conductor Software was used to log drop speed, latency to fall, and total distance over the course of the maximum 30‐min trial.

### 2.5 Brain Tissue Preparations

Procedures for preparation of samples and performance of ELISAs are detailed in the publications (Acker et al. 2013; Davidowitz et al. 2020; Forest et al. 2013). Three types of brain preparations were performed for assays to determine the levels of total self‐associated tau, Sarkosyl‐insoluble tau aggregates, and soluble tau in the heat stable (HS) fractions. For self‐associated tau, brain tissue from the cortex was homogenized in 10 volumes of cold Tris‐buffered saline, pH 7.4 containing protease and phosphatase inhibitors, cleared by low‐speed centrifugation at 14 000 × g for 10 min at 4°C, and stored at −80°C. To prepare Sarkosyl‐insoluble tau, the cleared homogenate was incubated for 10 min with 1% Sarkosyl, the insoluble tau was pelleted by ultracentrifugation at 200 000 × g for 30 min and washed with homogenization buffer. The pellet was suspended in 1 × Laemmli sample buffer and heated for 10 min at 100°C to dissociate the tau for analysis by ELISA. HS fractions of brain tissue were prepared by homogenization in 10 volumes of ice‐cold buffer (50 mM Tris, pH 7.5, 0.8 M NaCl, 5% β‐mercaptoethanol), and the cleared homogenate was heated at 95°C for 10 min and then centrifuged at 20 000 × g at 4°C for 10 min. Tau has the special characteristic of remaining soluble under these conditions, facilitating its purification in the supernatant/HS fraction. Supernatants were cooled and dialyzed against 50 mM Tris pH 7.5 with 1 mM EDTA, 0.1 mM PMSF.

### 2.6 ELISAs and Antibodies

Monoclonal antibodies (mAbs) used in the ELISAs were all developed, produced, and formatted for assays in the laboratory of Peter Davies, Ph.D., Director, Litwin‐Zucker Center for Alzheimer's Disease & Memory Disorders, FIMR. Pan‐tau antibodies mAb DA31 (epitope, amino acids 150–190 in 4R2N tau) and mAb DA9 (epitope, amino acids 102‐140). ELISAs were performed for total tau (capture Ab DA31, reporter Ab DA9HRP) and self‐aggregated tau by monoantibody tau ELISA (DA9‐DA9HRP) as described (Davidowitz et al. 2023; Forest et al. 2013).

### 2.7 Immunoblots

Thirty microgram of total protein from each brain lysate sample was loaded onto 4%–20% gradient polyacrylamide precast Criterion Gels (Bio‐Rad) with 0.1% final SDS under non‐reducing conditions, electrophoresed, and transferred onto PVDF membrane using the Transblot Turbo semi‐dry system (Bio‐Rad). The blots were ponceau‐stained followed by blocking in 5% non‐fat milk in PBST for 1 h at ambient temperature. The blots were then incubated overnight at 4°C in primary antibody HT7 (product # MN1000, Invitrogen) diluted 1:5000 in blocking buffer on a rocking platform. Following (3) 10‐min washes in blocking buffer, the blots were incubated for 1 h in goat‐anti‐mouse HRP conjugated (Jackson Immunochemicals) diluted 1:10 000 in blocking buffer at ambient temperature on a rocking platform. After several washes with decreasing concentrations of Tween‐20, the SuperSignal West Femto Maximum Sensitivity Substrate (ThermoFisher) was added, and chemiluminescent images were captured on the FluorChem R (Protein Simple) using movie mode exposures for quantification of chemiluminescent signal performed using AlphaView software (ProteinSimple). To measure the monomeric and aggregated tau from the blots, the luminescence counts for monomer, oligomer, and very high molecular weight (VHMW) tau were normalized to the total tau signal in the lane to enable comparison of the tau species between the samples on multiple blots.

### 2.8 Statistical Analyses

The study was powered by assuming a beta risk of 0.8 and an alpha risk of 0.05. The primary outcome was the reduction of insoluble tau aggregates in the brains of the treated mice compared to untreated mice with statistical significance. Results were expressed as mean ± standard error of the mean and presented as the percentage of the Vehicle group. All statistical analyses were performed using Graph‐Pad Prism 10.2 (GraphPad Software, San Diego, CA). Outliers, identified using the ROUT method with a false discovery rate (Q) set at 1%, are depicted as open squares in the figure. Normality was assessed beforehand using the D'Agostino & Pearson test and the Anderson‐Darling test. For data that met the assumptions of normality, multiple group comparisons were performed using the Brown‐Forsythe and Welch ANOVA tests, followed by the Dunnett T3 post hoc test (Tables S3 and S4). For comparisons between two groups, the two‐tailed Unpaired t‐test with Welch's correction was applied (Table S5). These parametric tests assume that the data are normally distributed and do not have equal variances or standard deviations. In cases where the data did not meet the assumptions for parametric tests, non‐parametric methods were employed. The Kruskal–Wallis test followed by Dunn's multiple comparisons test was used for multiple group comparisons (Tables S3 and S4), while the Mann–Whitney test was used for two‐group comparisons (Table S5). These non‐parametric tests do not assume a normal distribution. For multiple group comparisons, a P‐value less than 0.05 was considered statistically significant and indicated by the following symbols: * for *p* < 0.05, ** for *p* < 0.01, *** for *p* < 0.001, and **** for *p* < 0.0001. While for comparison between two groups, a *p* value less than 0.05 was considered statistically significant, and the exact *p* values were reported for these comparisons. The correlation of the levels of VHMW tau to the levels of total human tau in each mouse were plotted for each study group using exponential growth curves, and correlation analysis was performed with Spearman's rank correlation (*r*). Comparisons were considered statistically significant at an α‐level of *p* < 0.05.

### 2.9 Study Design

**FIGURE 1:**
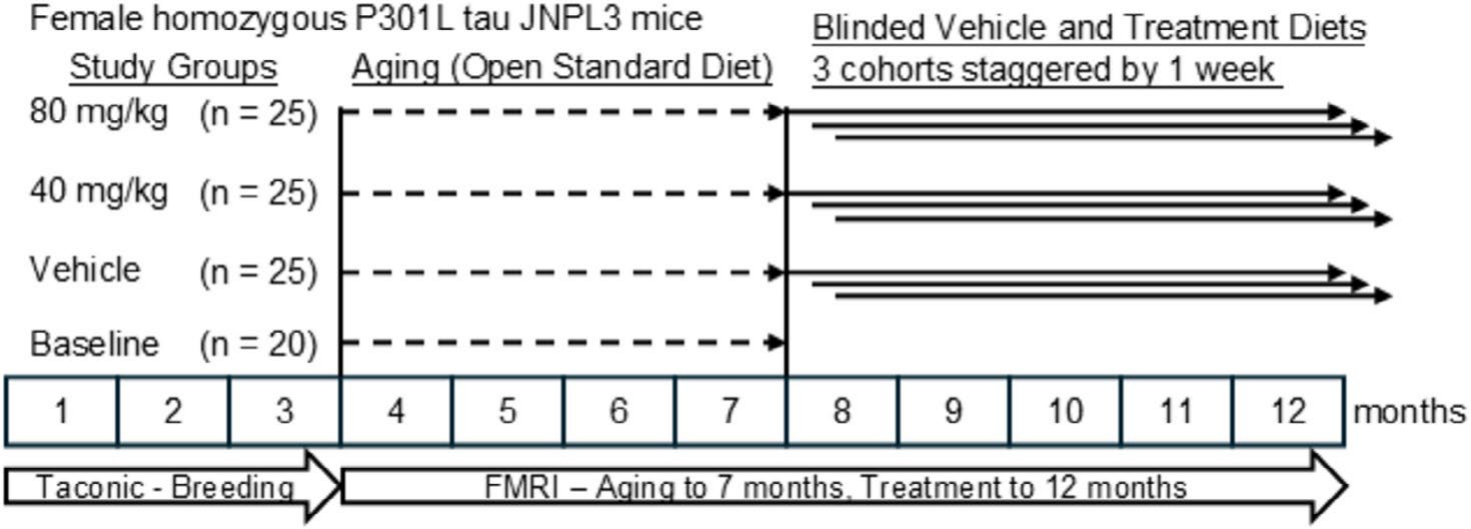
Schematic of study design. The study was independently performed at FIMR. Female homozygous P301L tau JNPL3 mice were bred at Taconic and aged at FIMR at 3–7 months. Vehicle and Treatment groups were evenly distributed in the three staggered cohorts. The Baseline group was harvested following the motor behavior assay at 7 months of age, and the motor behavior of the Vehicle and 40 and 80 mg/kg groups was assayed at the end of 5 months of treatment. Biochemical studies for tau aggregates were performed for all mice at the same time to minimize inter‐assay variability. Due to attrition during aging, samples for biochemical analysis were obtained from 19 mice in the vehicle group, 22 mice in the 40 mg/kg group, and 20 mice in the 80 mg/kg Treatment groups.

Mice were randomized using a block randomization design (Jimenez et al. 2023). Random assignment generators were used to assign animals born at the same time to different treatment arms in blocks until all the slots were filled. Mice were divided into four groups: a Baseline group (*n* = 20, harvested at 7 months‐of‐age), a vehicle group (*n* = 25, harvested at 12 months‐of age), and two treatment groups (40 and 80 mg/kg doses, *n* = 25 per group, harvested at 12 months‐of age). The vehicle and treatment groups were evenly divided into three cohorts. Mice treated with vehicle or drug were started in a group‐balanced manner over a 7‐day period to permit tissue collection exactly 5 months later with a practical number of mice each day. The diets for the Vehicle and Treatment diets were coded prior to delivery to FIMR so that investigators were blind to treatment group identity. No exclusion criteria were pre‐determined. There was attrition of 14 mice that was not test article related: 6 from the Vehicle group, 3 from the 40 mg/kg group, and 5 from the 80 mg/kg group. There were no drug‐related side effects of this compound during the entire length of the study. Samples for biochemical analysis were obtained from the remaining mice. Data were unblinded after the analyses were completed. The study design is shown in Figure 1

## 3 Results

### 3.1 Oral Treatment of Aged P301L Tau JNPL3 Mice With OLX‐07010 Inhibited the Accumulation of Tau Aggregates

**FIGURE 2:**
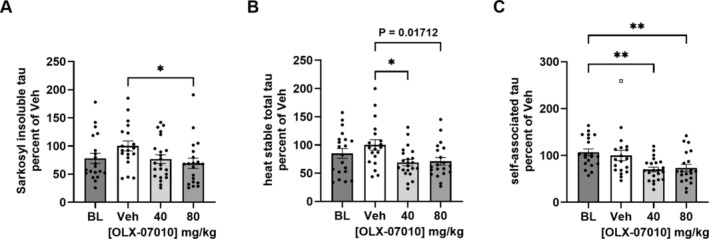
Analysis of total and aggregated tau protein in the cortex. Quantification of tau was performed by ELISAs that detect both the endogenous mouse tau and the exogenously expressed human tau construct. The four groups in the study were Baseline (BL; *n* = 20), sacrificed at 7 months, and Vehicle (Veh; *n* = 19), 40 mg/kg (*n* = 22), and 80 mg/kg (*n* = 20) groups sacrificed at 12 months. Sarkosyl Insoluble total tau (A) and total tau (B) in the heat stable fraction were quantified by ELISA formatted with mAb DA31 for capture and mAb DA9‐HRP for reporter. Self‐associated tau (C) was determined by mono‐Ab ELISA formatted with mAb DA9 for capture and mAb DA9‐HRP for reporter. Results for BL and treatment groups are shown as a percentage of the mean value of the Vehicle group (% Veh). Outliers, identified using the ROUT method (*Q* = 1%), are shown as open squares. For group comparisons, the significance level of *p* < 0.05 is considered statistically significant and indicated as * for *p* < 0.05 or ** for *p* < 0.01.

ELISAs for total tau determined the combined levels of the human 4R0N P301L tau construct and endogenous murine tau in mature mice in the cortex (Figure 2, Tables S3 and S4). Sarkosyl insoluble tau increased 22% from the baseline to the Vehicle group, but treatment caused a 25% decrease in the 40 mg/kg dose group and a 30% decrease in the 80 mg/kg dose group compared to the Vehicle group, indicating both a linear dose response and statistical significance at the high dose (Figure 2A). Total levels of tau in the heat‐stable fraction increased from baseline to the Vehicle group by 15%, but the levels of tau were decreased by ~30% in both treatment groups (Figure 2B) with statistical significance at both doses. During the treatment period, the level of self‐associated tau in cleared lysate did not vary between the mean of the Baseline and Vehicle groups, but treatment caused a 30% reduction of self‐associated tau with the highest statistical significance between baseline and treatment groups (Figure 2C).

**FIGURE 3:**
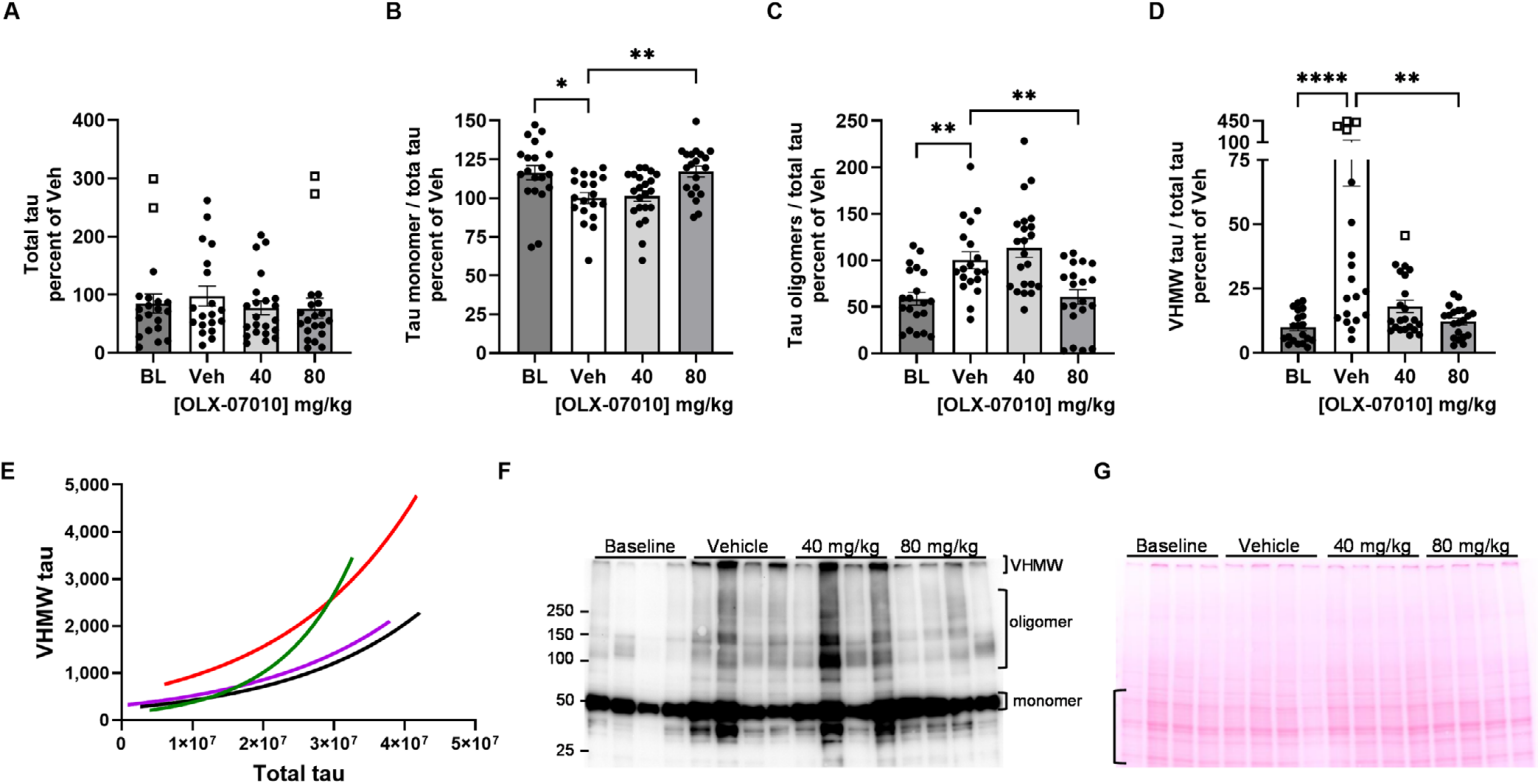
Immunoblot analysis of tau species using mAb HT7. Immunoblot analysis of tau was performed with mAb HT7 specific for the human tau construct using total protein tissue lysates from cortex. Overall human tau signal from each lane was normalized to Ponceau staining in that lane (A). The signal for tau monomer (B), tau oligomers (C) or very high molecular weight (VHMW) tau (D) was normalized to the total tau signal in each lane for each sample. Results for BL and treatment groups are shown as a percentage of the mean value of the Vehicle group (% Veh). Outliers, identified using the ROUT method (*Q* = 1%), are shown as open squares. For group comparisons, significance levels are indicated as follows: *p < 0.05, **p < 0.01, and ****p < 0.0001. For pairwise comparisons, *p* < 0.05 is considered significant, with exact values reported. Exponential growth curves representing the correlation of the levels of VHMW tau (E) to the levels of total human tau in each mouse were plotted for each study group and overlayed to show the effect of 40 mg/kg (green) and 80 mg/kg (purple) treatment relative to the control Baseline (black), and Vehicle (red) groups. (R squared values for correlation exponential curves, N): Red (0.6483, 19); Green (0.7134, 22); Purple (0.7454, 20); Black (0.6359, 20). A representative mAb HT7 immunoblot image (F) indicates the regions of the image that were quantified for monomer, oligomer, and VHMW tau. The signal for monomer, oligomer and VHMW tau at the top of the gel was normalized to the total tau signal in the lane to enable comparison of the tau species between the samples on multiple blots. A representative image of a Ponceau stained membrane has a bracket (G) indicates the region quantified by imageJ (Rasband 1997‐2018) for the normalization of the total tau signal in each lane (A). All immunoblots in this dataset, including the ones shown here, are also presented in Figure S2.

To specifically study the effects of treatment on the size distribution of aggregates of the human P301L tau construct and to confirm the results of the ELISAs, we performed immunoblots with the monoclonal antibody HT7 that specifically binds human tau independently of tau phosphorylation (Figure 3, Tables S3 and S4). Total protein lysates prepared with non‐denaturing buffer were run using conditions to minimize disruption of tau oligomers and very high molecular weight (VHMW) aggregates. The samples were not heated nor treated with a reducing agent, and only 0.1% SDS detergent was used to prepare the protein samples. Total human tau was quantified for the entire lane and normalized to Ponceau stained protein in that lane; there were no statistically significant differences between the study groups (Figure 3A). The amount of monomer, oligomer, and VHMW tau relative to total tau in each lane was compared by study group (Figure 3B–E). This approach also normalized values for the samples in different lanes and on different blots. The Baseline group and high dose treatment group had higher levels of tau monomer compared to the Vehicle group (Figure 3B), whereas the baseline and high dose group had lower levels of tau oligomer (Figure 3C) and VHMW (Figure 3D) species compared to the Vehicle group with statistical significance. The levels of tau species in the low dose treatment group appear similar to the levels of tau species in the Vehicle group when the data are presented in bar graphs.

The variable expression of the human tau construct between JNPL3 mice obscured the effect of treatment between groups when the data was analyzed independently of this variable. To determine the effect of the inhibitor over the range of tau expression within each group, the levels of monomer and aggregated tau species were correlated to the total tau signal from each lane of the immunoblots to derive a single exponential growth curve for each group. The curves for the VHMW tau were plotted and overlaid to directly compare the Baseline (black) and Vehicle (red) groups, and the 40 mg/kg (green) and 80 mg/kg (purple) treatment groups (Figure 3F). The 40 mg/kg dose was sufficient to maintain VHMW tau at baseline levels in mice expressing lower levels of the human tau construct, whereas in mice with higher levels of expression of the human tau construct, the dose was insufficient for preventing the VHMW tau from reaching levels in the Vehicle group (Figure 3F, green and red curves). In contrast, the 80 mg/kg dose was effective at maintaining the VHMW tau at baseline across the spectrum of expression of the human tau construct in the mice (Figure 3E, purple and black curves). Together, these data demonstrated a dose effect that was dependent on the level of tau transgene expression in the animals. Images of an immunoblot and its corresponding Ponceau‐stained membrane are shown to indicate the regions selected for quantification (Figure 3F,G).

### 3.2 Treatment of the Aged Mice Partially Rescued Motor Impairment

**FIGURE 4:**
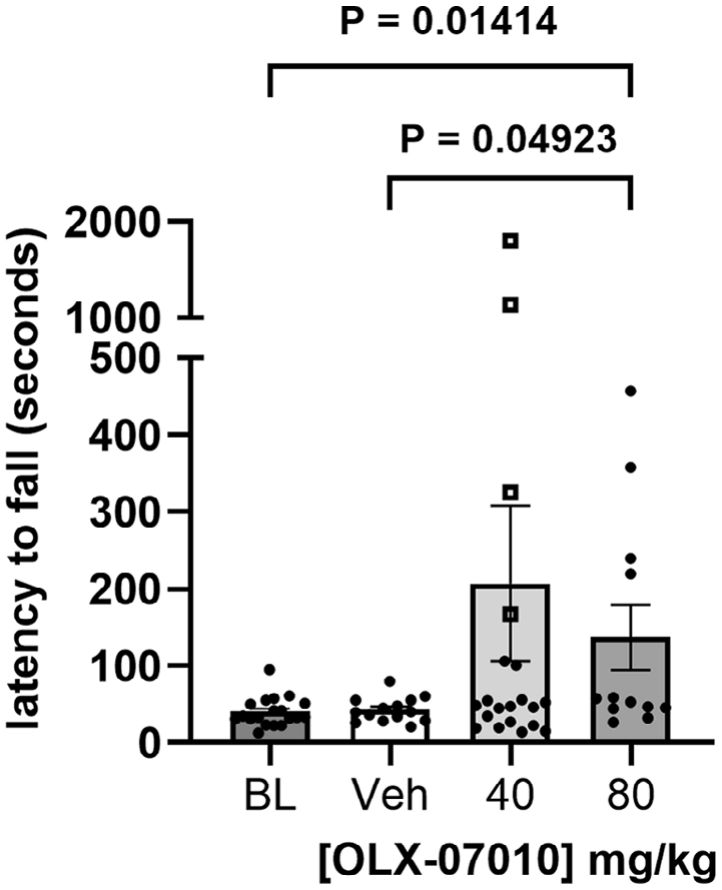
Evaluation of motor coordination. Cross‐sectional evaluation of motor coordination was performed with a Rotarod assay. The mice were evaluated prior to sacrifice, the Baseline group at 7 months, and the Vehicle, 40 and 80 mg/kg Treatment groups at 12 months. Data presented as mean ± SEM. Outliers, identified using the ROUT method (*Q* = 1%), are shown as open squares. For comparison between selected two groups, *p* < 0.05 is considered significant, with exact values reported.

JNPL3 mice develop weakened paw strength and hindlimb paralysis as they age past 9 months (Lewis et al. 2000). To evaluate the effect of treatment on motor function, Rotarod assays were performed on the Baseline group at 7 months and on the Vehicle and treatment groups at 12 months. At both 7 and 12 months, the untreated JNPL3 mice had poor performance on the Rotarod, falling typically in less than 60 s. Mice treated with the high dose had improved performance with increased latency to fall off the rod with statistical significance in pairwise comparisons to the Baseline and Vehicle groups (Figure 4; Table S5).

### 3.3 Serum Levels of OLX‐07010 Increased Linearly With Dose

**FIGURE 5:**
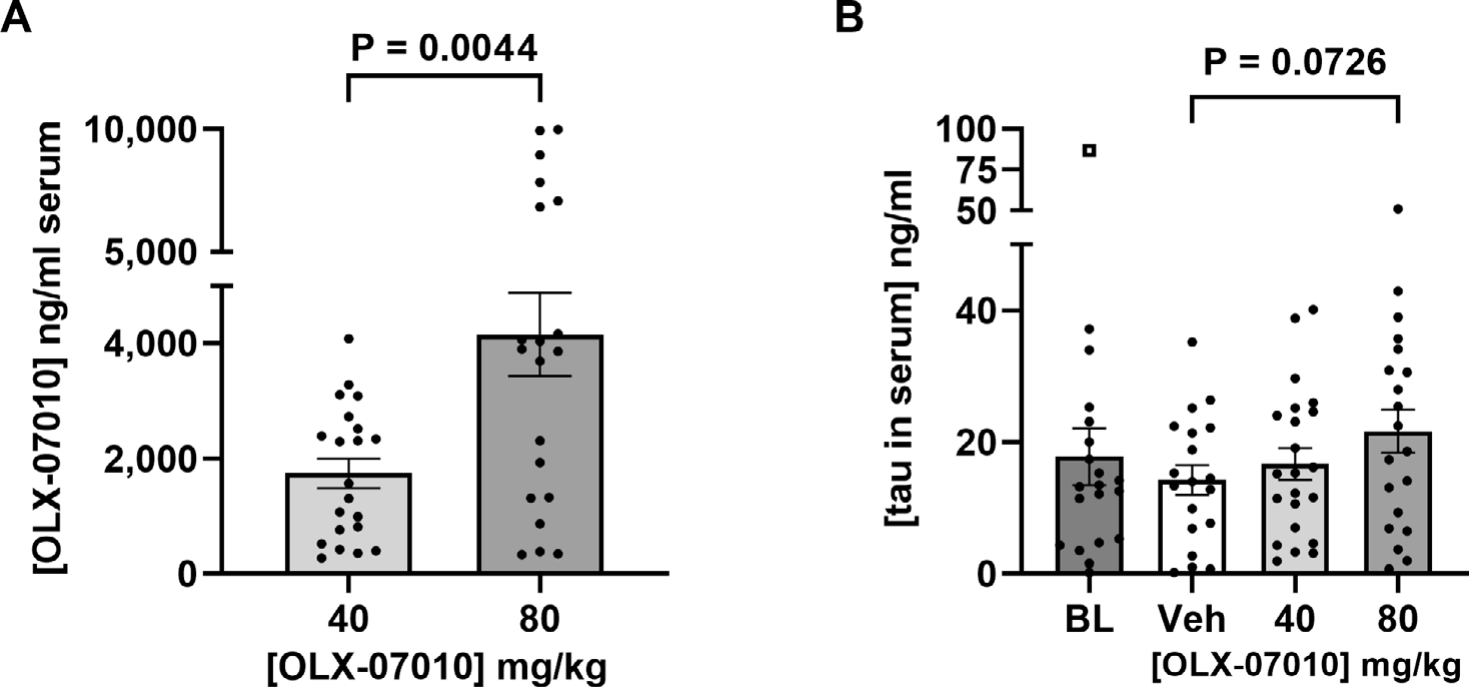
Serum levels of OLX‐07010 and tau protein. (A) Quantification of compound levels in mice sera was performed by LC/MS/MS with an Agilent 6400 LC/Triple Quad. Mice sera was processed from whole blood collected terminally in the middle of the dark cycle. (B) Heat treatment of sera at acidic pH was used to remove interfering antibodies from the samples to enable quantification of tau by ELISA (d'Abramo et al. 2016). Data presented as mean ± SEM. Outliers, identified using the ROUT method (*Q* = 1%), are shown as open squares. For comparison between selected two groups, *p* < 0.05 is considered significant, with exact values reported.

The concentration of OLX‐07010 serum was collected at the study endpoint to confirm the treatment groups received the correct doses, to evaluate the mean concentration and its relationship to dose. Doubling of the dose in the feed led to about a two‐fold increase in the mean concentration in the serum (Figure 5A). The scatter in the data was expected as OLX‐07010 was administered in the diet that was fed ad libitum. Mice were sacrificed in the middle of their 12‐h dark cycle during which time they ate most of their diets formulated with the compound. Analysis of total tau in the serum by ELISA showed a trend to increase in total tau in the high‐dose group compared to the Vehicle group.

## 4 Discussion

A therapeutic study design was used to evaluate treatment with OLX‐07010 on the accumulation of tau aggregates in an aged mouse model of 4R tauopathy, homozygous female P301L tau JNPL3 mice (Figure 1). Female JNPL3 mice develop pathology earlier and more severely than males (Buccarello et al. 2017; Koppel et al. 2019) and were therefore chosen to characterize the effect of treatment by 12 months of age. The primary endpoint of the study was achieved with the high‐dose treatment that reduced the level of Sarkosyl‐insoluble tau in the cortex to Baseline with statistical significance in comparison to the Vehicle group (Figure 2A). The levels of total tau in the heat‐stable fraction of the cortex were also reduced in the low and high Treatment groups with statistical significance compared to Vehicle (Figure 2B). The levels of self‐associated tau were similar in untreated mice in the Baseline (7 months) and Vehicle (12 months) groups but were decreased in both Treatment groups with statistical significance compared to Baseline. Together, these ELISA data suggest that treatment inhibited the accumulation of tau protein and its soluble and insoluble aggregates in aged mice with tau pathology prior to the start of treatment.

To better understand the relationship between the level of expression of the human tau construct in individual mice to the levels of their tau aggregates and the effect of treatment on them, tau monomer and aggregated species samples were resolved using minimally denaturing SDS‐PAGE and immunoblot to detect the human tau construct (Figure 3F,G; Figures S3 and S4). The results suggest that there was an exponential relationship between the levels of high molecular weight tau aggregates relative to the total level of tau present in the samples. Furthermore, the effective dose was dependent on the level of total tau in the samples such that the 40 mg/kg dose was effective in mice expressing low levels of tau but ineffective in mice expressing higher levels of tau. Using this approach, the variability of tau expression in the mice enabled us to evaluate the dose‐target relationship and the pharmacodynamic response within the study groups (Figure 3E). In addition to the biochemical readouts, treatment of the aged mice partially rescued motor impairment in the Rotarod assays (Figure 4). The motor deficits in JNPL3 mice are associated with the accumulation of insoluble tau in the spinal cord, axonal degeneration in anterior spinal roots, and neurogenic atrophy in muscle (Lewis et al. 2000). The phenotype of this mouse model does not include the development of cognitive behavioral deficits and therefore precludes tests for learning and memory impairment to study the treatment effect. The effect of treatment on both motor function and on tau aggregation, along with the absence of drug‐related side effects of this compound during the entire length of the study, highlights the therapeutic potential of OLX‐07010.

This chronic treatment study had some limitations. Although this transgenic model may be representative of tau aggregation in inherited 4R tauopathies, this model is not representative of tau aggregation in Alzheimer's disease, in which there are no mutations in tau associated with the disease and in which both 3R and 4R tau aggregate. Additionally, the JNPL3 mice do not overexpress the human amyloid precursor protein (APP) and do not form the amyloid beta that causes inflammation and hyperphosphorylation of tau, and it would therefore be beneficial to evaluate OLX‐07010 expressing both proteins (Lewis et al. 2001). Although immunocytochemistry of brain sections was part of the planned study, the tissue could not be processed and was lost due to the absence of access to the laboratory on consecutive days during the COVID‐19 pandemic and is planned for future studies. Direct analysis of motor neuron loss in the spinal cord by immunostaining with an antibody for NeuN in the spinal cord of JNPL3 mice (Okuda et al. 2015) would have provided valuable information in the evaluation of a neuroprotective effect of treatment with OLX‐07010.

Treatment was administered orally by a diet formulated with the compound and the doses were approximated based on average mouse body weight and daily consumption of feed provided ad libitum. Although administration in the diet is easier to perform and less stressful for the mice, especially for a chronic treatment study, it is not amenable for evaluating the pharmacokinetics of the compound. A study in mice performed with a single daily dose by oral gavage would provide a better understanding of the relationship between the pharmacodynamic and pharmacokinetic parameters that could translate to a clinical study. An acute treatment study using oral gavage may also result in a more rapid output of pharmacodynamic data along with a better understanding of exposure in the brain.

The Rotarod test was used to evaluate the motor coordination of the mice using cross‐sectional comparisons between the performance of the Baseline group at 7 monthsof age and the Vehicle and Treatment groups at 12 months. Unexpectedly, the Rotarod performance of the Baseline cohort in this study at 7 months of age was similar to the Vehicle group at 12 months of age, suggesting that there was no progression in the deterioration of motor behavior. One of the limitations of this study was that the behavioral analysis was cross‐sectional, not longitudinal. There is phenotypic variability between individual mice and between cohorts of mice in this model. A longitudinal study of Rotarod performance would have enabled a better assessment of the progressive phenotype by comparing the percent change in individual mice between the groups. Our future studies will include longitudinal evaluation of behavior. Although the 40 mg/kg group had a higher mean latency to fall, this difference was not statistically significant. In contrast, the 80 mg/kg group showed significant improvement compared to Baseline and Vehicle groups (Figure 4; Table S3). Additionally, the 40 mg/kg group had four outliers with high values, identified using the ROUT method (*Q* = 1%), which increased variability and affected statistical significance. Longitudinal evaluation of percent change in performance would also help mitigate the confounding effects of biological variability.

Recent comprehensive reviews of the therapeutic landscape of tauopathies (Cummings et al. 2023; Lane‐Donovan and Boxer 2024) catalog the multiple approaches and clinical programs targeting tau. There are no approved drugs directly targeting tau and only about 20 that are currently in clinical trials. The most common therapeutic approaches are immunotherapies targeting extracellular tau and drugs targeting tau aggregation. Small molecule drugs have numerous advantages over immunotherapeutics for tauopathies because they can penetrate the brain, entering individual neurons and extracellular vesicles to directly interfere with tau aggregation. A small molecule tau aggregation inhibitor, hydromethylthionine mesylate, a derivative of methylene blue, was selected to dissociate tau filaments, and Phase 3 trials failed to achieve its clinical endpoints (Gauthier et al. 2016). In contrast, OLX‐07010 was selected to inhibit the first step in the aggregation process, tau self‐association, and is in early preclinical development. There exists a strong need for cost‐effective, oral, small molecule, brain‐penetrant disease‐modifying therapeutics for AD and related tauopathies. The value of an oral small molecule tau treatment is that it represents a more cost‐effective, brain‐penetrant therapeutic option with lower patient burden compared with antibody approaches targeting tau. An effective inhibitor of tau self‐association has the potential for utility in the treatment of numerous rare tauopathies with unmet needs. A small molecule approach allows for ease of administration globally and serves as a complementary therapy.

## Author Contributions

**E. J. Davidowitz:** conceptualization, methodology, data curation, investigation, supervision, project administration, funding acquisition, writing – original draft, formal analysis, writing – review and editing, validation. **P. Lopez:** methodology, writing – review and editing, formal analysis, data curation, writing – original draft. **D. Patel:** writing – review and editing, formal analysis, data curation. **H. Jimenez:** methodology, writing – review and editing, data curation. **A. Wolin:** methodology. **J. Eun:** methodology, data curation. **L. Adrien:** methodology. **J. Koppel:** methodology, data curation, formal analysis, project administration, writing – review and editing. **D. Morgan:** writing – review and editing. **P. Davies:** conceptualization, methodology, supervision, resources, project administration, formal analysis, data curation. **J. G. Moe:** conceptualization, supervision, investigation, funding acquisition, writing – review and editing, project administration, reporting.

## Conflicts of Interest

E.D., P.L., D.P., and J.M. are full‐time employees of Oligomerix Inc. and own stocks in the company. D.M. consults with In Med, MindImmune, SynapsDx, and Hesperos on projects unrelated to this work. The remaining authors declare no conflicts of interest.

## Supporting information


Data S1.

## Data Availability

The data for this publication can be accessed using the following unique digital object identifier (DOI): https://doi.org/10.5061/dryad.1g1jwsv75.
