# Supplementary material for: Therapeutic Treatment With OLX‐07010 Inhibited Tau Aggregation and Ameliorated Motor Deficits in an Aged Mouse Model of Tauopathy
Source: J Neurochem. 2025 Mar 7;169(3):e70025. doi: 10.1111/jnc.70025 (PMC11886763; doi:10.1111/jnc.70025)
Supplement: Supplementary file 1 — Data S1. [file JNC-169-0-s001.docx]

**Title**

Therapeutic treatment with OLX-07010 inhibited tau aggregation and ameliorated motor deficits in an aged mouse model of tauopathy

**Authors**

Davidowitz EJ^1, 2^, Lopez P^2^, Patel D^2^, Jimenez H^3^, Wolin A^3^, Eun J^3^, Adrien L^3^, Koppel J^3^, Morgan D^4^, Davies P and Moe JG^1, 2^

**Affiliations**

^1^Oligomerix, Inc., White Plains, NY, United States of America.

^2^Oligomerix, Inc., Bronx, NY, United States of America.

^3^The Litwin-Zucker Research Center for the Study of Alzheimer's Disease, The Feinstein Institutes for Medical Research, Northwell Health, Manhasset, NY, United States of America.

^4^Department of Translational Neuroscience and the Alzheimer's Alliance Michigan State University Grand Rapids Michigan USA.

**Corresponding Author:** Eliot J. Davidowitz

Address: Oligomerix, Inc., 2 Westchester Park Drive, Suite 208, White Plains, NY 10604

Telephone numbers: cell 917-692-2521; office 212-568-0365

Email address of the person to whom proofs and reprint requests should be addressed: [edavidowitz@oligomerix.com](mailto:edavidowitz@oligomerix.com)

**Alphabetical list of abbreviations used in the text**:

AD, Alzheimer's disease; AUC (area under curve); BL, baseline; FIMR, Feinstein Institutes for Medical Research; HRP, horse radish peroxidase; HS, heat stable; htau, human tau; IACUC, Institutional Animal Care and Use Committee; mAb, monoclonal antibody; RRID, Research Resource Identifier; Veh, vehicle; VHMW, very high molecular weight

**Supplementary Materials and Method**

**Pharmacokinetic study**

The in-life part of the pharmacokinetic study was conducted at Bayside Biosciences, Inc. (Santa Clara). Female CD-1 mice (Charles River Lab) aged 7-8 weeks with body weight between 20 to 31 g were acclimated for 6 days before the experiment. The mice were given 200 mg/kg of OLX-07010 in 1xPBS via oral (P.O.) administration. At 30 min, 1, 2, 4, 6, and 8 hours post dosing, two mice were anesthetized with Isoflurane. Blood sample was collected from cardiac puncture followed by brain perfusion with 10 ml cold 1xPBS. The perfused brain tissue was collected/weighed and frozen down in dry ice. The blood samples in EDTA tubes were centrifuged at 5,000 rpm at 4 ^o^C for 10 minutes. The plasma was transferred to individual wells in a 96-well microtiter plate and stored on dry ice. The samples were shipped on dry ice to Quintara Discovery for analysis.

At Quintara Discovery, the plasma samples were thawed and mixed well. The Brain tissues were thawed on ice and homogenized with two times the volume of chilled water using bead based TissueLyser II. Calibration standards of OLX-07010 were prepared by spiking the compound into the blank plasma or brain homogenate at a series of concentrations. Twenty μL of the samples or standards were treated with 100 μL of internal standard (verapamil) containing organic solution, methanol: acetonitrile 5:95 (v/v) to precipitate the protein. The mixtures were vortexed for 15 minutes and centrifuged at 4000 rpm at 4 ^o^C for 15 minutes. Fifty μL of the supernatant was transferred to an injection plate and mixed with 100 μL of 0.1% formic acid in water for the analysis on LC-MS/MS. Positive electrospray ionizations under the multiple-reaction-monitoring (MRM) mode was utilized for the detection of the analyte. The data analysis software used was Analyst 1.6.2 (AB Sciex).

**Supplemental Figure 1.** Mean plasma/brain concentration-time course of OLX-07010 after PO administration of 200 mg/kg dose to CD-1 mice (n=2).

| **Time  (hr)** | **[OLX-07010] in CD-1 mice** | | | | | | | | | |
| --- | --- | --- | --- | --- | --- | --- | --- | --- | --- | --- |
|  | **Brain (ng/g)** | | | | | **Plasma (ng/mL)** | | | | |
|  | **Mice 1** | **Mice 2** | **Mean** | **SD** | **CV%** | **Mice 1** | **Mice 2** | **Mean** | **SD** | **CV%** |
| **0.5** | 1503 | 3720 | 2610 | 1570 | 60 | 12000 | 24900 | 18500 | 9120 | 49.4 |
| **1** | 4950 | 3360 | 4160 | 1120 | 27.1 | 28100 | 37300 | 32700 | 6510 | 19.9 |
| **2** | 3180 | 3240 | 3210 | 42.4 | 1.32 | 24900 | 28900 | 26900 | 2830 | 10.5 |
| **4** | 1842 | 1887 | 1860 | 31.8 | 1.71 | 21000 | 13700 | 17400 | 5160 | 29.8 |
| **6** | 2313 | 1014 | 1660 | 919 | 55.2 | 22700 | 9990 | 16300 | 8990 | 55 |
| **8** | 1413 | 1500 | 1460 | 61.5 | 4.22 | 10900 | 13400 | 12200 | 1770 | 14.5 |

**Supplemental Table 1.** Concentrations of OLX-07010 in individual samples plotted as the mean concentration curves in Supplemental Figure 1.

| **Matrix** | **Dose** | **T_max_** | **C_max_** | **T_1/2_** | **MRT_last_** | **MRT_inf_** | **AUC_last_** | **AUC_inf_** |
| --- | --- | --- | --- | --- | --- | --- | --- | --- |
|  | **(mg/kg)** | **(hr)** | **(ng/g)** | **(hr)** | **(hr)** | **(hr)** | **(hr*ng/g) or (hr*ng/mL)** | **(hr*ng/g) or (hr*ng/mL)** |
| **Brain** | 200 | 1 | 4160 | 5.58 | 3.38 | 8.42 | 17700 | 29500 |
| **Plasma** | 200 | 1 | 32700 | 5.67 | 3.53 | 8.5 | 154000 | 253000 |

**Supplemental Table 2.** Summary of the pharmacokinetic (PK) evaluation of OLX-07010 in CD-1 mice after P.O. administration of 200 mg/kg dose. The PK parameters were evaluated using non-compartmental model by Phoenix™ WinNonlin® software (Pharsight Corporation).

**Supplemental Figure 2. Immunoblots for human tau.** Immunoblots for human tau were performed using mAb HT7 (A-E), and the corresponding Ponceau stained membranes are shown below each immunoblot (F-J). The immunoblots in 2C and 2H are also presented in Figure 3F and 3G of the main manuscript. For immunoblots 1 and 2, lanes 1-4 represent (Baseline); lanes 5-8 represent (Vehicle); lanes 9-13 represent (40 mg/kg) and lanes 14-17 represent (80 mg/kg). For immunoblots 3 and 4: lanes 1-4 represent (Baseline); lanes 5-8 represent (Vehicle); lanes 9-12 represent (40 mg/kg) and lanes 13-16 represent (80 mg/kg). For immunoblots 3 and 4, lanes 1-4 represents (Baseline); lanes 5-8 represents (Vehicle); lanes 9-13 represents (40 mg/kg) and lanes 14-17 represents (80 mg/kg). For immunoblot 5, lanes 1-4 represent (Baseline); lanes 5-7 represents (Vehicle); lanes 8-11 represents (40 mg/kg) and lanes 12-15 represents (80 mg/kg). Detection of primary antibody binding was performed with goat anti mouse secondary Ab conjugated to HRP with Femto chemiluminescence substrate (Pierce, Thermofisher). The FluorChemR™ (Protein Simple, San Jose, CA) imager was used to capture images, and quantification of chemiluminescent signal was performed using AlphaView software (Protein Simple). The protein concentration in each lysate was quantified by BCA assay (Pierce, ThermoFisher) and 30 μg protein was loaded in each well.

| **FIGURE** | **NORMALITY TEST OUTCOME** | **TEST** | **TEST STATISTICS** | **P VALUE** |
| --- | --- | --- | --- | --- |
| **2A** | Passed | Kruskal-Wallis | H (Treatment groups, Total values) = 7.786 (4, 81) | 0.0506 |
| **2B** | Failed | Brown-Forsythe ANOVA | F* (DFn, DFd) = 3.456 (3.000, 67.02) | 0.0212 |
| **2C** | Failed | Kruskal-Wallis | H (Treatment groups, Total values) = 16.47 (4, 81) | 0.0009 |
| **3A** | Failed | Kruskal-Wallis | H (Treatment groups, Total values) = 1.201 (4, 81) | 0.7528 |
| **3B** | Passed | Brown-Forsythe ANOVA | F* (DFn, DFd) = 6.012 (3.000, 71.10) | 0.0010 |
| **3C** | Passed | Brown-Forsythe ANOVA | F* (DFn, DFd) = 10.64 (3.000, 72.50) | <0.0001 |
| **3D** | Failed | Kruskal-Wallis | H (Treatment groups, Total values) = 19.32 (4, 81) | 0.0002 |
| **4** | Failed | Kruskal-Wallis | H (Treatment groups, Total values) = 6.121 (4, 81) | 0.1059 |
| **5B** | Failed | Kruskal-Wallis | H (Treatment groups, Total values) = 2.923 (4, 81) | 0.4036 |

**Supplemental Table 3.** List of the Test statics and respective significance value (P) for group-wise comparison performed in Figures 2, 3, 4 and 5.

| **FIGURE** | **COMPARISIONS** | **NORMALITY TEST OUTCOME** | **TEST** | **TEST**  **STATISTICS** | **P VALUE** |
| --- | --- | --- | --- | --- | --- |
| **2A** | Veh vs. BL | Failed | Dunn’s | Z = 1.9270 | 0.1619 |
|  | Veh vs. 40 mg/kg | Failed | Dunn’s | Z = 1.9170 | 0.1655 |
|  | Veh vs. 80 mg/kg | Failed | Dunn’s | Z = 2.6870 | ✱  0.0216 |
| **2B** | Veh vs. BL | Passed | Dunnett's T3 | t = 1.1780,  df = 36.62 | 0.5654 |
|  | Veh vs. 40 mg/kg | Passed | Dunnett's T3 | t = 2.8640,  df = 30.21 | ✱  0.0223 |
|  | Veh vs. 80 mg/kg | Passed | Dunnett's T3 | t = 2.5090,  df = 33.39 | ✱  0.0500 |
| **2C** | Veh vs. BL | Failed | Dunn’s | Z = 1.0070 | >0.9999 |
|  | BL vs. 40 mg/kg | Failed | Dunn’s | Z = 3.4160 | ✱✱  0.0038 |
|  | BL vs. 80 mg/kg | Failed | Dunn’s | Z = 3.1720 | ✱✱  0.0091 |
| **3A** | Veh vs. BL | Failed | Dunn’s | Z = 0.2563 | >0.9999 |
|  | Veh vs. 40 mg/kg | Failed | Dunn’s | Z = 0.6224 | >0.9999 |
|  | Veh vs. 80 mg/kg | Failed | Dunn’s | Z = 1.0260 | 0.9149 |
| **3B** | Veh vs. BL | Passed | Dunnett's T3 | t = 2.8200,  df = 34.83 | ✱  0.0232 |
|  | Veh vs. 40 mg/kg | Passed | Dunnett's T3 | t = 0.2482,  df = 38.61 | 0.0223 |
|  | Veh vs. 80 mg/kg | Passed | Dunnett's T3 | t = 2.5090,  df = 33.39 | ✱✱  0.0043 |
| **3C** | Veh vs. BL | Passed | Dunnett's T3 | t = 3.6060, df = 34.07 | ✱✱  0.0029 |
|  | Veh vs. 40 mg/kg | Passed | Dunnett's T3 | t = 0.9756,  df = 39.00 | 0.7004 |
|  | Veh vs. 80 mg/kg | Passed | Dunnett's T3 | t = 3.3570,  df = 35.97 | ✱✱  0.0073 |
| **3D** | Veh vs. BL | Failed | Dunn’s | Z = 4.1910 | ✱✱✱✱  <0.0001 |
|  | Veh vs. 40 mg/kg | Failed | Dunn’s | Z = 2.0110 | 0.1330 |
|  | Veh vs. 80 mg/kg | Failed | Dunn’s | Z = 3.1360 | ✱✱  0.0051 |

**Supplemental Table 4.** List of the Test statics and respective significance value (P) for post hoc analyses of Group-wise multiple comparisons in Figures 2 and 3.

| **FIGURE** | **COMPARISIONS** | **NORMALITY TEST OUTCOME** | **TEST** | **TEST**  **STATISTICS** | **P VALUE** |
| --- | --- | --- | --- | --- | --- |
| **4** | BL vs. 80 mg/kg | Failed | Mann Whitney | U = 54 | 0.0141 |
|  | Veh vs. 80 mg/kg | Passed | Welch’s t | t = 2.2040,  df = 11.22 | 0.0492 |
| **5A** | 40 mg/kg vs.  80 mg/kg | Passed | Welch’s t | t = 3.1500,  df = 23.57 | 0.0044 |
| **5B** | Veh vs. 80 mg/kg | Passed | Welch’s t | t = 1.8550,  df = 33.21 | 0.0726 |

**Supplemental Table 5.** List of the Test statics and respective significance value (P) for two-tailed unpaired comparison between two selected groups in Figures 4 and 5.
